# Comparative ileal amino acid digestibility and growth performance in growing pigs fed different level of canola meal

**DOI:** 10.1186/s40781-015-0055-3

**Published:** 2015-06-03

**Authors:** Kwangyeol Kim, Akshat Goel, Suhyup Lee, Yohan Choi, Byung-Jo Chae

**Affiliations:** Department of Animal Resources Science, College of Animal Life Sciences, Kangwon National University, Chuncheon, 200-701 Republic of Korea

**Keywords:** Protein source, Canola meal, Digestibility, Performance, Economic importance, Weanling-growing pigs

## Abstract

The digestibility of different vegetable protein sources were investigated and the effects of supplementing canola meal (CM) as partial inclusions were studied in growing pigs, to determine the performance parameters and its economic importance. In Exp. 1, four pigs (average initial BW = 15.4 ± 0.35 kg, 5 weeks of age) fitted with simple T-cannula at terminal ileum, were fed four diets following repeated 4 × 4 Latin square design having adoption period of 7 days. Diet 1 was Nitrogen free diet containing corn starch. Diets 2, 3, and 4 were the basal diet supplemented with soybean meal (SBM), rapeseed meal (RSM), and domestic CM respectively. The AID of crude protein was decrease in RSM in comparison to SBM supplementation. The AID of Dietary indispensable amino acids (DIAA) such as Lys, Meth, Pha, and dispensable amino acid Ala, Pro, Asp were decreased (P < 0.05) in RSM supplemented diets. The SID of DIAA does not differ but the SID of Asp was higher (P < 0.05) in RSM and CM diets while SID of Pro was lower (P < 0.05) in RSM in comparison to SBM supplemented diets. In Exp. 2, 192 growing pigs (average initial BW 24.76 ± 2.55 kg) were randomly allotted to four dietary treatments with increasing levels of CM i.e. 0, 3.75, 7.50, and 11.25 % respectively. Diets were fed in meal form for 35 days. Increasing CM levels in diets had no effects (P > 0.05) on growth performance and apparent total tract digestibility (ATTD) of nutrients and energy. Total weight gain, total feed intake, and feed cost per kg weight gain were not affected by increasing levels of CM in diets but total feed cost (TFC) per pigs was linearly reduced (26.463 to 25.674; P < 0.05). Broadly, the AID, and SID of amino acid was reduced in RSM but was not effected in CM in comparison to SBM supplemented pigs. Moreover, increasing levels of CM in pigs diet had no effect on the ATTD and performance but TFC per pig was reduced. Thus CM inclusion of up to 11.25 % in diets can be used for reducing the production cost in growing pigs without any negative effect.

## Background

Cost of pork production is mainly dependent on feed and accounts for almost 70 % of the total cost [1, 2]. Consistent increase in the price of soybean in the last few years has substantially increased the feed cost [3, 4]. To overcome this issue, other cheap protein sources are being investigated, that can be used as a substitute of soybean but their selection and optimization is necessary. Few of the most common replacement are rapeseed and canola. Rapeseed meal (RSM) is an economical dietary protein source that acts as a second most widely traded protein ingredient for world protein meal production [5]. Moreover canola is the product of the genetic modifications of rapeseed and sometimes also known as double low variety [6]. The RSM contains about 40 % crude protein (on DM basis) and about similar or even better amino acids (AA) contents, but its nutrient value depends on the kind of seed, cultural environment, and processing methods [7, 8]. Similarly canola meal (CM) is also famous for its rich protein content and AA profile [9]. Being a residual product of oil extraction process and its easy availability due to increasing use in the biofuel industry, these products are becoming popular alternative protein source for swine production. However high erucic acid and glucosinolates content is the major limiting factor with rapeseed meal that causes change in the thyroid tissue, and inclusion at high levels in diets for long periods may affect growing-finishing performance and thyroid hypertrophy [10, 11]. Drawback with canola meal is its higher level of fiber content that decreases its digestibility [9]. To get the optimum growth, it is important to design the diets on the bases of digestibility i.e. apparent ileal digestibility (AID) and standardized ileal digestibility (SID). Moreover, to make the diets economical without having any negative effect on the performance, it is important to consider the inclusion levels of ingredients in diets. To select the best economical protein source according to their digestibility and to optimize its concentration in the feed of growing pigs, the present research was conducted with RSM and CM for partially substituting it with SBM in growing pigs.

In order to prepare the economical feed composition with optimum performance, the current study was conducted with three different protein supplemented diets i.e. SBM, RSM, and CM. Apparent ileal digestibility (AID) and standardized ileal digestibility (SID) were studied to compare the AA digestibility. Further, the most digestible protein source was further supplemented as inclusions at different concentration in the growing pigs’ diet to determine its effects on performance and economic viability.

## Methods

Two experiments were designed and conducted on weanling and growing pigs (Landrace × Yorkshire × Duroc) at the facility of Kangwon National University Farm Chuncheon Republic of Korea to study the effect of different protein diets and graded level of CM inclusions on AID, SID of AA, performance, and its economic efficiency in weanling and growing pigs. Both experiments were approved (approval number: KW-141111-2) and swine were cared according to the guidelines of the Institutional Animal Care and Use Committee of Kangwon National University, Chuncheon, Republic of Korea.

### Digestibility trial

After one day of fasting, four weanling pigs, average initial BW 15.4 ± 0.35 kg, were fitted with a simple T-cannula at the terminal ileum according to the method suggested by Walker et al. [12]. Semi-purified diets were formulated with each protein sources to be tested, i.e. with N-free diet, SBM, RSM, and CM respectively (Table 1). N-free diet was used to study the basal endogenous losses. These diets were fed according to a repeated 4 × 4 Latin square design. Vitamins and minerals were supplemented to meet or exceed NRC [13], standards for pigs. All diets contained 0.25 % chromic oxide as the digestibility marker. Each pig was fed 900 gm of feed in three times a day. Each treatment diets were fed for 7 days duration followed by 3 days collection of digesta samples. Second treatment was conducted after 7 days adoption period. Similar routine was followed until all the pigs received all the treatments. The collected samples were immediately frozen at −80 °C, freeze dried (Samwon Inc., Korea), ground in a 1 mm-mesh Wiley Mill, and stored in a refrigerator until analysis.

**Table 1:** Formula and chemical composition of experimental diets for ileal digestibility trials

Item	N-free	Soybean meal	Rapeseed meal	Canola meal
Ingredients (%)				
Corn starch	56.50	44.79	32.34	37.94
Soybean meal (44 %)	-	52.24	-	-
Rapeseed meal (35 %)	-	-	65.57	-
Canola meal (38 %)	-	-	-	59.78
Sucrose	20.00	-	-	-
Glucose	20.00	-	-	-
Choline chloride (50 %)	0.05	0.05	0.05	0.05
TCP	1.68	1.13	0.65	0.74
Limestone	0.62	0.64	0.24	0.34
Salt	0.30	0.30	0.30	0.30
Mineral premix^1^	0.30	0.30	0.30	0.30
Vitamin premix^2^	0.30	0.30	0.30	0.30
Chromic oxide	0.25	0.25	0.25	0.25
Total	100.00	100.00	100.00	100.00
Chemical composition (%)				
ME (kcal/kg)	3,509	3,391	2,904	3,125
CP	-	23.00	23.00	23.00
Calcium	0.70	0.70	0.70	0.70
Available phosphorus	0.32	0.32	0.32	0.32
Lysine	-	1.44	1.31	1.18
Met + Cys	-	0.66	1.08	0.76

^1^ Supplied per kg diet: 150 mg Fe, 96 mg Cu, 72 mg Zn, 46.49 mg Mn, 0.9 mg I, 0.9 mg Co, 0.336 mg Se

^2^ Supplied per kg diet: 10,000 IU Vit A, 2,500 IU Vit D3, 50 IU Vit E, 1.5 mg Vit K3, 1.5 mg Vit B1, 5 mg Vit B2, 3 mg Vit B6, 0.025 mg Vit B12, 15 mg pantothenic acid, 35 mg niacin, 0.15 mg biotin, 1 mg folic acid

Proximate analyses were done by following the methods of AOAC [14]. Gross energy was measured by using bomb calorimeter (Model 1261, Parr Instrument Co., Molin, IL), and chromium with an automated spectrophotometer (Shimadzu, Japan) following the methods of Fenton and Fenton [15], respectively. After the acid hydrolysis in 6 N HCL at 105 °C for 24 h, concentrations of AA were analyzed using a HPLC (Waters 486, USA). Analysis of sulfur containing AA was done after cold performic acid oxidation overnight with subsequent hydrolysis as suggested by Moore [16].

### Performance trial

Total of 192 pigs (Landrace × Yorkshire × Duroc) with an average initial body eight (BW) 24.76 ± 2.55 kg at 8 week of age were used in a 35-days growth assay. The pigs were allotted to four treatments composed of four pens in each treatment with twelve pigs in each pen. Each 2.8 × 5 m pen was equipped with a self-feeder and nipple waterer to allow ad libitum consumption of feed and water. The animals were weighed and feed intake was also determined. The average final BW of the pigs was 49.31 kg. The dietary treatments included a basal diet supplemented with 0, 3.75, 7.50, and 11.25 % CM substrate respectively. Diets for experiment were formulated to contain 3,350 (kcal/kg) ME, 18 % CP and 0.98 % lysine. Vitamins and minerals were supplemented in all diets and all diets met or exceeded the estimated nutrient requirements suggested for growing pigs by the National Research Council [13].

The pigs were weighed individually and feed consumption of each pen was measured at the end of the experiments. Growth performance in terms of average daily gain (ADG), average daily feed intake (ADFI) and F:G was calculated during the feeding trial. The apparent total tract digestibility (ATTD) of energy and nutrients were evaluated using 0.25 % chromic oxide (an inert indigestible indicator) in each diet from d 28 to d 35 of each experiment and ATTD of DM, GE, and CP were determined by collecting fecal grab samples during last 4 days from the floor of each pen. Pooled fecal samples within pan were then dried in a forced air drying oven at 60 °C for 72 h, and ground in a Wiley mill (Thomas Model 4 Wiley Mill, Thomas Scientific, Swedesboro, NJ) using a 1-mm screen and used for chemical analysis.

Each sample was analyzed in triplicate for DM (Method 930.15), CP (Method 990.03), ash (Method 942.05), Ca, and P (Method 985.01) according to the methods of AOAC [14]. Gross energy of diets and excreta were measured using a bomb calorimeter (Model 1261, Parr Instrument Co., Molin, IL), while chromium concentrations were determined with an automated spectrophotometer (Shimadzu, Japan) according to the procedure described by Fenton and Fenton [15].

Calculation of feed cost (FC) was based on the price of ingredients. Feed cost per kg body weight gain (FCG) and total feed cost (TFC) was calculated as follows:

$$ \mathrm{F}\mathrm{C}\mathrm{G} = \mathrm{T}\mathrm{F}\mathrm{I}\times \mathrm{F}\mathrm{C}/\mathrm{T}\mathrm{W}\mathrm{G} $$

$$ \mathrm{T}\mathrm{F}\mathrm{C} = \mathrm{F}\mathrm{C} \times \mathrm{T}\mathrm{F}\mathrm{I} $$

Where, TFI = total feed intake and FC = feed cost

TWG = total weight gain per pig (kg).

### Statistical analyses

Data collected were subjected to statistical analysis using the General Linear Model (GLM). Procedure of SAS [17] was used as complete randomized block design. Orthogonal polynomials were used to evaluate linear and quadratic effects of dietary domestic CM supplementation. The treatments were the main effects. The pens were the experimental units for all analysis but for ileal digestibility each pig was the experimental unit while probability values of ≤ 0.05 were considered significant.

## Results

### Digestibility trial

The pigs remained healthy and consumed their feed daily throughout the experiment. No symptoms of disease were seen in any of the pig throughout the experiment.

The chemical composition of the diets is presented in Table 1. Three different protein sources were used in this experiment and one nitrogen free diet was used to measure basal endogenous losses. The level of CP was 23 % and was kept constant in all the three protein source containing diets. Proximate analysis (Table 2) revealed that RSM had lower CP values in comparison to its counterparts. Similarly total mean and sub mean values of AA were also lower in RSM.

**Table 2:** Proximate and amino acid composition of the protein sources used for ileal digestibility

Item (%)	Soybean meal	Rapeseed meal	Canola meal
DM	86.33	87.51	88.63
CP	44.96	34.22	37.77
Ash	4.95	6.52	6.29
CF	5.60	8.54	9.50
Ca	0.40	1.15	0.66
P	0.42	0.76	1.03
NDF	12.06	22.29	25.06
ADF	8.30	14.19	16.18
Amino acid			
Dietary indispensable amino acids			
Arginine	3.27	2.16	2.13
Histidine	1.19	0.91	0.97
Isoleucine	2.04	1.25	1.36
Leucine	3.47	2.27	2.51
Lysine	2.83	1.57	2.09
Methionine	0.61	0.65	0.74
Phenylalanine	2.34	1.36	1.44
Threonine	1.83	1.40	1.58
Tryptophan	0.55	0.33	0.37
Valine	2.10	1.57	1.72
Sub-mean	20.23	13.47	14.91
Dietary dispensable amino acids			
Alanine	1.95	1.40	1.57
Aspartic acid	5.31	2.21	2.53
Cystine	0.64	0.88	0.91
Glutamic acid	8.47	6.17	6.34
Glycine	1.94	1.70	1.80
Proline	2.28	2.20	2.29
Serine	2.37	1.42	1.58
Tyrosine	1.51	0.87	0.95
Sub-mean	24.47	16.85	17.97
Total-mean	44.70	30.32	32.88

The AID of CP and AA of the experimental diets are presented in Table 3. Few of the AA and CP has shown significant responses (P < 0.05) in the AID of different protein source supplemented diets. The AID of CP was lower in RSM but does not differ in CM in comparison to SBM. Amino acid digestibility values were lower in diets supplemented with RSM for DIAA (Lys, Meth and Pla), and DDAA (Ala, Asp, and Pro) in comparison to SBM.

**Table 3:** Apparent ileal digestibility of protein sources in pigs

Item (%)	Soybean meal	Rapeseed meal	Canola meal	SEM^1^	*p*-value
CP	71.46^a^	68.10^b^	70.51^ab^	0.98	0.015
Dietary indispensable amino acids					
Arginine	81.50	80.28	83.53	1.24	0.087
Histidine	79.58	77.02	78.10	1.10	0.167
Isoleucine	80.68	76.62	78.04	1.48	0.062
Leucine	81.09	79.77	80.12	1.06	0.622
Lysine	78.69^a^	73.03^b^	76.46^ab^	1.80	0.016
Methionine	80.02^a^	76.72^b^	79.30^ab^	1.10	0.020
Phenylalanine	80.73^a^	77.62^b^	79.26^ab^	1.06	0.041
Threonine	74.05	71.99	75.12	1.18	0.075
Tryptophane	73.04	70.35	72.61	2.64	0.713
Valine	73.16	71.64	72.46	1.26	0.665
Dietary dispensable amino acids					
Alanine	72.37^a^	69.83^b^	71.67^ab^	0.82	0.013
Aspartic acid	73.80^a^	70.57^b^	71.40^ab^	1.14	0.039
Cystine	72.06	70.72	72.01	2.02	0.855
Glutamic acid	80.14	77.39	79.95	1.08	0.052
Glycine	72.81	71.50	72.27	0.84	0.491
Proline	79.26^a^	77.11^b^	78.63^ab^	0.74	0.040
Serine	75.57	74.06	74.10	0.86	0.297
Tyrosine	75.77	72.83	74.02	1.58	0.348

^ab^ Values with different superscripts of the same row are significantly differ (p<0.05)

^1^ Standard error of means

The SID of AA for the experimental diets of different protein sources is presented in Table 4. The SID of DIAA does not differ in the dietary treatments but the SID of DDAA like Asp was higher (P < 0.05) in RSM and CM diets while SID of Pro was lower (P < 0.05) in RSM in comparison to SBM supplemented group.

**Table 4:** Standardized ileal digestibility of protein sources in pigs

Item (%)	Soybean meal	Rapeseed meal	Canola meal	SEM^1^	*p*-value
Dietary indispensable amino acids					
Arginine	87.00	88.66	92.02	1.27	0.055
Histidine	89.55	90.16	90.42	1.22	0.877
Isoleucine	89.15	90.54	90.82	2.35	0.867
Leucine	86.59	88.23	87.77	0.82	0.380
Lysine	84.67	83.88	84.60	1.54	0.924
Methionine	87.69	83.97	85.66	1.27	0.171
Phenylalanine	86.57	87.74	88.81	1.02	0.343
Threonine	83.87	84.93	86.57	1.00	0.211
Tryptophan	85.79	91.77	91.69	2.09	0.121
Valine	81.38	82.72	82.56	1.80	0.850
Dietary dispensable amino acids					
Alanine	80.48	81.22	81.82	0.86	0.567
Aspartic acid	80.02^b^	85.65^a^	84.56^a^	1.30	0.031
Cystine	82.73	78.54	79.57	2.70	0.542
Glutamic acid	83.91	82.61	85.03	0.76	0.135
Glycine	90.05	91.32	90.97	0.95	0.632
Proline	86.35^a^	84.52^b^	85.74^ab^	0.43	0.038
Serine	83.16	86.82	85.56	1.12	0.116
Tyrosine	86.01	90.74	90.40	3.15	0.520

^ab^ Values with different superscripts of the same row are significantly differ (p<0.05)

^1^ Standard error of means

### Performance trial

All the diets contained similar CP (18 %) and ME (3,350 kcal/kg) with increasing inclusion levels of CM 0, 3.75, 7.50, and 11.25 % (Table 5). Increasing inclusion of CM from 0 to 11.25 % did not affect BW, ADG, ADFI, and F:G (Table 6) in growing pigs.

**Table 5:** Ingredient and chemical composition of experimental diets

Item	Canola meal (%)			
	0	3.75	7.50	11.25
Cost ($, kg)	0.455	0.451	0.448	0.445
Ingredient (%)				
Corn	66.54	66.42	65.72	64.81
Soybean meal (44 %)^1^	27.34	23.88	20.86	17.88
Domestic canola meal (37 %)	0.00	3.75	7.50	11.25
Animal fat	2.50	2.50	2.58	2.74
Choline-chloride (50 %)	0.08	0.08	0.08	0.08
_L_-lysine HCl (78 %)	0.31	0.33	0.34	0.35
_DL_-methionine (99 %)	0.09	0.07	0.06	0.04
_L_-threonine (98.5 %)	0.07	0.07	0.07	0.07
_L_-tryptophan (10 %)	0.26	0.30	0.32	0.34
TCP	0.91	0.94	0.98	1.01
Limestone	1.20	0.96	0.79	0.73
Salt	0.25	0.25	0.25	0.25
Mineral premix^2^	0.20	0.20	0.20	0.20
Vitamin premix^3^	0.20	0.20	0.20	0.20
Phytase	0.05	0.05	0.05	0.05
Total	100.00	100.00	100.00	100.00
Chemical composition (%)				
ME (kcal/kg)	3,350	3,350	3,350	3,350
CP	18.00	18.00	18.00	18.00
Calcium	0.75	0.75	0.75	0.75
Available phosphorus	0.26	0.26	0.26	0.26
SID lysine	0.98	0.98	0.98	0.98
SID met + cys	0.55	0.55	0.55	0.55
SID threonine	0.59	0.59	0.59	0.59
SID tryptophan	0.17	0.17	0.17	0.17

^1^ Soybean meal was replaced by domestic canola meal (SBM: 687 ₩/kg; CM: 480 ₩/kg)

^2^ Supplied per kg diet: 150 mg Fe, 96 mg Cu, 72 mg Zn, 46.49 mg Mn, 0.9 mg I, 0.9 mg Co, 0.336 mg Se

^3^ Supplied per kg diet: 10,000 IU Vit A, 2,500 IU Vit D3, 50 IU Vit E, 1.5 mg Vit K3, 1.5 mg Vit B1, 5 mg Vit B2, 3 mg Vit B6, 0.025 mg Vit B12, 15 mg pantothenic acid, 35 mg niacin, 0.15 mg biotin, 1 mg folic acid

**Table 6:** Effects of supplementation of canola meal on growth performance in growing pigs

Item	Canola meal (%)				SEM^1^	*p*-value	
	0	3.75	7.50	11.25		Linear	Quadratic
Initial BW (kg)	24.44	24.37	24.33	24.32	0.14	0.572	0.879
Final BW (kg)	49.81	49.41	49.17	48.82	0.38	0.076	0.950
ADG (g)	725	715	710	700	10.24	0.102	0.996
ADFI (g)	1,584	1,577	1,571	1,573	14.76	0.625	0.775
F:G	2.18	2.21	2.21	2.25	0.02	0.196	0.822

^1^ Standard error of means

Few limiting amino acids were added in the feed for maintaining the amino acid content. The digestibility of nutrients was not effected in increasing CM supplementation and there was no variation (P > 0.05) in DM, GE, CP, and Ash (Table 7) content of growing pigs.

**Table 7:** The effect of different supplemental levels of canola meal on nutrient digestibility in pigs

Item	Canola meal (%)				SEM^1^	*p*-value	
	0	3.75	7.50	11.25		Linear	Quadratic
DM	80.09	79.59	79.17	79.02	0.70	0.330	0.926
GE	79.67	79.41	78.93	78.06	0.81	0.183	0.718
CP	73.92	73.81	73.52	73.19	0.66	0.468	0.879
Ash	40.18	38.68	38.39	38.32	0.88	0.169	0.436

^1^ Standard error of means

There was a linear decrease (P < 0.05) in the total feed cost in the pigs with increasing inclusion of CM in diets (Table 8). However no difference (P > 0.05) was observed in the FC ($/kg), TWG (kg/pig), TFI (kg/pig), and FCG ($/kg wt. gain) of the finishing pigs.

**Table 8:** Effects of level of supplementation of canola meal on the production cost in pigs

Item	Canola meal (%)				SEM^1^	*p*-value	
	0	3.75	7.50	11.25		Linear	Quadratic
FC ($/kg)	0.455	0.451	0.448	0.445			
TWG (kg/pig)	25.37	25.04	24.84	24.50	0.36	0.102	0.997
TFI (kg/pig)	55.45	55.20	55.01	55.09	0.50	0.623	0.779
TFC ($/pig)	26.463	26.118	25.825	25.674	0.29	0.047	0.724
FCG ($/kg wt. gain)	1.043	1.044	1.040	1.048	0.01	0.883	0.796

^1^ Standard error of means

Dietary supplementation of increasing CM levels had no effects (P > 0.05) on growth performance and ATTD of nutrients and energy. Total weight gain, total feed intake, and feed cost per kg weight gain were not affected by increasing levels of CM in diets but total feed cost per pigs was reduced (P < 0.05).

## Discussion

### Effects of different protein source on digestibility performance in weaning pigs

The inclusion of SBM in pig diets as a protein supply is getting costlier, making researchers to look for better alternatives. Rapeseed meal and canola meal are the first few economical choices after SBM and contribute to almost 12.4 % of the world protein meal production [5]. Rapeseed meal has high crude protein content than soybean meal and contains about 30-40 % crude protein on fed bases and 35.96 to 44.75 % on dry matter basis [11, 18]. In the present study, proximate analysis revealed (Table 2) similar value of CP (34.22 %) for RSM, however it was lower in comparison to its counterparts. The level of CP was kept constant (Table 1) in all the three experimental diets containing different protein source. However, RSM supplemented diets had lower AID values of CP in comparison to SBM (Table 3). This is in line with the earlier studies of Li et al. [19] as they reported AID values of CP and most of the AA were significantly lower in rapeseed meal than soybean meal. This could be due to the lower proximate mean and sub mean values of AA in RSM supplemented diets. The other reasons behind this might be the lower percent of CP on fed bases or the quality of RSM as the concentration of CP and AA in canola and rapeseed products varies. Further it depends on many factors such as varieties, environmental factors, seed composition, and amount of residual oil and carbohydrates in the meal [6, 10].

The values of AID and SID of DIAA in SBM treatment were in similar range to the recent reports of Upadhaya and Kim [20]. Inclusion of 2 to 10 % canola in cornstarch-based soybean meal diets increases the ileal digestibility of most of the indispensable amino acids [21]. However, in the present study, CM treatment had nearby values of AID (Table 3) and SID (Table 4) of amino acid and did not differ with SBM or RSM supplementations. Lysine is considered the first limiting amino acid in most diets and plays an important role in metabolism [22, 23]. Decrease in the AID of Lys, in RSM inclusion might be due to the direct absorption of Lys by the intestine for protein synthesis and other metabolic processes [24]. Methionine is required for growth and maintenance of body protein [25]. Proline is important for differentiation, multiple biochemical, and physiological processes in cells and serves as a major AA for the synthesis of polyamines that improves growth, development, and morphology of small-intestinal in weanling piglet [26, 27]. In this experiment, AID of Meth, and SID of Pro was significantly decreased in RSM inclusion in comparison to SBM. This could be due to the lower digestion of Meth and Pro in RSM and the limited ability of pigs to synthesize proline [28] that may further affect growth performance.

Consistent decrease in the amino acid digestibility in RSM inclusion in pig’s diet might be due to the higher content of fibers that causes poor digestibility, increase endogenous secretions, and decrease hydrolysis and absorption of nutrients [10, 29]. Further, anti-nutritional factors such as glucosinolates could be the other reason that usually affects young pigs the most [30].

### Effects of increasing level of canola meal on the performance of growing pigs

The second experiment was conducted to evaluate the effect of inclusion of different levels of CM on the performance and economic benefits in growing pigs. Canola is a genetically modification of traditional varieties of rapeseeds to obtain low level of anti-nutritional factors such as glucosinolates [6]. Studies conducted on chicken had shown that increasing levels of CM in diets resulted in decreasing the cost of production [31, 32]. However economic benefits in pig are yet to be considered. Previous studies revealed that swine diets should be formulated on the basis of true or standardized amino acid digestibility [33]. Therefore, mixing ratio was created by applying the standardized ileal digestibility (SID) that has shown good efficiency on swine.

Increasing level of CM supplementation of up to 200 gm/kg in diets or 25 % inclusion decreases the ATTD of DM and CP in pigs [34, 35]. However in the current study, the maximum dietary inclusions of CM is 11.25 %, that is almost half in comparison to the above study therefore no variation (P > 0.05) was observed in ATTD of DM, CP, and GE (Table 7).

The present study was conducted on growing pigs with the maximum of 11.25 % of CM inclusions in the diets to minimize the effects of antinutritional factors. Dietary inclusion of increasing levels of CM does not have any effect on TWG, TFI, and FCG per kg weight gain. This is in line with the earlier studies of King et al. [36] as they reported no adverse effects of up to 20 % solvent-extracted canola meal on the performance of pigs. This might be due to the use of glucosinolates below the tolerance limit in diets. In similar type of studies conducted on growing-finishing pigs, other workers suggested even higher level of up to 20 to 30 % dietary supplementation of canola products [37, 38]. However, using 16.2 % canola meal in diets fed to finishing pigs (60 to 120 kg BW) resulted in reduced ADG compared with pigs fed the control diet without canola meal due to the increasing impact of glucosinolates with time [11].

Decrease (P < 0.05) in the price of total feed cost per pigs (Table 8) was observed in current study. This is in accordance with the earlier studies of Seneviratne et al. [39] where they suggested inclusion of canola meal in swine diets reduces the feed costs per unit of BW gain without affecting carcass and fat quality. These are the positive sign for pig growers for using CM in diets without affecting nutrient digestibility and growth performance in pigs.

## Conclusions

We therefore conclude that RSM inclusion in diets had lower digestibility of few amino acid in comparison to SBM but digestibility of CM inclusion in diets of weanling pigs were not affected. Inclusion of up to 11.25 % CM in diets had no effect on the performance and nutrient digestibility of the growing pigs. However the TFC ($/pig) was reduced linearly. Thus CM can be used up to 11.25 % for replacing SBM in diets of growing pigs for reducing the feed cost in pig production.

## Abbreviations

SBM: Soybean meal
RSM: Rapeseed meal
CM: Canola meal
CP: Crude protein
ADG: Average daily gain
DM: Dry matter
ADFI: Average daily feed intake
DIAA: Dietary indispensable amino acids
DDAA: Dietary dispensable amino acids
