# Erratum to: Comparative ileal amino acid digestibility and growth performance in growing pigs fed different level of canola meal

**DOI:** 10.1186/s40781-015-0075-z

**Published:** 2015-11-30

**Authors:** Kwangyeol Kim, Akshat Goel, Suhyup Lee, Yohan Choi, Byung-Jo Chae

**Affiliations:** Department of Animal Resources Science, College of Animal Life Sciences, Kangwon National University, Chuncheon, 200-701 Republic of Korea

## Erratum

The original version of this article [1] did not include an Acknowledgments section. This has been rectified with this Erratum. Please find the Acknowledgments section below.
